# Circulating osteogenic proteins are associated with coronary artery calcification and increase after myocardial infarction

**DOI:** 10.1371/journal.pone.0202738

**Published:** 2018-08-23

**Authors:** Antonio E. Pesaro, Marcelo Katz, Marcel Liberman, Carolina Pereira, Cristovão L. P. Mangueira, Ana E. Z. de Carvalho, Karina S. Carvalho, Cesar H. Nomura, Marcelo Franken, Carlos V. Serrano

**Affiliations:** Hospital Israelita Albert Einstein, São Paulo, SP, Brazil; Brigham and Women's Hospital, Harvard Medical School, UNITED STATES

## Abstract

**Background:**

Coronary artery calcification (CAC) and atherosclerotic inflammation associate with increased risk of myocardial infarction (MI). Vascular calcification is regulated by osteogenic proteins (OPs). It is unknown whether an association exists between CAC and plasma OPs and if they are affected by atherothrombotic inflammation. We tested the association of osteogenic and inflammatory proteins with CAC and assessed these biomarkers after MI.

**Methods:**

Circulating OPs (osteoprotegerin, RANKL, fetuin-A, Matrix Gla protein [MGP]) and inflammatory proteins (C-reactive protein, oxidized-LDL, tumoral necrosis factor-α, transforming growth factor [TGF]-β1) were compared between stable patients with CAC (CAC ≥ 100 AU, n = 100) and controls (CAC = 0 AU, n = 30). The association between biomarkers and CAC was tested by multivariate analysis. In patients with MI (n = 40), biomarkers were compared between acute phase and 1–2 months post-MI, using controls as a baseline.

**Results:**

MGP and fetuin-A levels were higher within individuals with CAC. Higher levels of MGP and RANKL were associated with CAC (OR 3.12 [95% CI 1.20–8.11], p = 0.02; and OR 1.75 [95% CI 1.04–2.94] respectively, p = 0.035). After MI, C-reactive protein, OPG and oxidized-LDL levels increased in the acute phase, whereas MGP and TGF-β1 increased 1–2 months post-MI.

**Conclusions:**

Higher MGP and RANKL levels associate with CAC. These findings highlight the potential role of these proteins as modulators and markers of CAC. In addition, the post-MI increase in OPG and MGP, as well as of inflammatory proteins suggest that the regulation of these OPs is affected by atherothrombotic inflammation.

## Introduction

The presence and progression of coronary artery calcification (CAC) are associated with increased risk of future coronary heart disease events [1],[2]. The increase in atherosclerotic and atherothrombotic inflammation, as indicated by the circulating levels of inflammatory proteins, is associated with adverse cardiovascular events in primary prevention individuals, as well as in acute myocardial infarction (MI) patients. [3, 4]

Importantly, pathological mechanisms underlying CAC progression are still under debate, and up to this moment, there is no medical treatment to attenuate CAC. Previous results indicate that multiple osteogenic proteins (OPs) act as modulators of atherosclerotic calcification. [5]. The osteoblastic dedifferentiation of vascular smooth muscle cells is mediated by OPs, such as bone morphogenetic proteins and the receptor activator of nuclear factor kappa-B ligand (RANKL). Also, osteoprotegerin (OPG), fetuin-A and matrix Gla protein (MGP) may counter-regulate vascular calcification, all of which act by inhibiting hydroxyapatite formation and deposition. [6, 7] Few clinical studies addressed whether plasma levels of OPs can be associated with CAC. In spite of the known inhibitory effect of OPG on vascular calcification, plasma OPG levels were found to be associated with CAC in unselected healthy subjects and in diabetic patients. [8, 9] Another study on community-living individuals showed that plasma fetuin-A levels were inversely associated with the severity of CAC.[10] Studies designed to investigate correlations between plasma MGP levels and CAC had discordant results which can possibly be accounted for by the use of different laboratory assays.[11–14]

Additionally, the interrelationship between CAC and atherosclerotic inflammation remains controversial. Yet, experimental studies demonstrated that inflammatory pathways can modulate vascular calcification and OPs expression. Oxidized (ox)-LDL and oxidative stress stimulate osteoblastic dedifferentiation of vascular smooth muscle cells.[15, 16]. Activated T-lymphocytes and in endothelial cells express RANKL and OPG [17]. In addition, OPG is also stimulated by multiple inflammatory mediators, which also augment the local expression of adhesion molecules and leukocyte infiltration in the vessel wall. [18] On the other hand, clinical evidence did not confirm the association between atherosclerotic inflammation and CAC. Although higher levels of inflammatory proteins, such as interleukin-6, oxLDL and the monocyte chemotactic protein-1, were shown by some authors to be associated with CAC, a meta-analysis failed to confirm this association.[19–21] Also, one large clinical study did not find an association between baseline plasma high-sensitivity C-reactive protein (hs-CRP) and CAC progression. [22]

Finally, in patients with acute MI, the prognostic role of baseline circulating inflammatory and osteogenic proteins was clinically well established, but few studies followed the levels of OPs after the acute and subacute phases.[23–25]. Moreover, whether OPs expression is affected by increased atherothrombotic inflammation after MI remains largely unknown. The existing hypothesis is that the increased inflammatory response in patients with acute MI could initiate osteochondrogenic signaling, that would be reflected as a transient oscillation of specific OPs.

Therefore, in the present study, we aimed to test the association between selected circulating OPs and inflammatory proteins with CAC in stable patients, and to assess these biomarkers in MI patients in the acute phase and after the subacute phase (i.e. 1–2 months post-MI).

## Patients and methods

### Study population

The study was approved by the local ethic committee (Sistema Gerenciador de Projeto de Pesquisa—SGPP—Hospital Israelita Albert Einstein, São Paulo, SP, Brazil. Approval number 1666–12) according to guidelines of the Declaration of Helsinki. All patients provided informed written consent. From March 2014 to November 2015, 170 patients were prospectively enrolled. Stable patients without known coronary artery disease, who underwent ambulatory coronary computed tomography, as indicated by the patient’s assistant medical staff, were selected according to CAC score: (1) CAC patients (CAC score ≥ 100 Agatston units [AU], n = 100); and (2) Control patients (CAC score = 0 AU, n = 30). Exclusion criteria for these patients were previous MI, previous coronary angioplasty or coronary artery bypass graft surgery, recent (< 3 months) non-cardiac surgical procedures, chronic kidney disease (creatinine > 1.5 mg/dL), cancer, chronic obstructive pulmonary disease, current infections, vasculitis, collagen diseases, chronic inflammatory diseases and immunosuppressive treatment. After providing informed written consent, the patients were asked to fill out a brief questionnaire and to have a blood sample drawn. Blood was obtained immediately after performing coronary computed tomography or during early clinical follow-up (<1 month) after coronary computed tomography.

Additionally, 46 patients hospitalized for acute MI were enrolled. Six patients declined to participate during follow-up period and were excluded from the analysis. Patients with MI were evaluated during the acute phase (3±1 days post-MI) and 1–2 months post-MI (46±17 days post-MI). Acute MI was defined according to international guidelines criteria [26]: typical rise and gradual fall of biochemical markers of myocardial necrosis (troponin or creatine kinase-MB) with at least one of the following: 1) ischemic symptoms, 2) development of pathologic Q waves on the electrocardiogram (ECG), 3) ECG changes indicative of ischemia (ST-segment elevation or depression), or 4) coronary artery intervention (e.g., coronary angioplasty). Exclusion criteria for MI patients were: previous coronary artery bypass graft surgery, recent (< 3 months) MI, recent (< 3 months) cardiac or non-cardiac surgical procedures, chronic kidney disease (creatinine > 1.5 mg/dL), cancer, chronic obstructive pulmonary disease, current infections, vasculitis, collagen diseases, inflammatory chronic diseases and immunosuppressive treatment. Acute MI patients who consented to participate in the study were asked to fill out a questionnaire. Blood samples were collected at this point (acute phase) and at the medical follow-up after approximately 1–2 months.

### Assessment of CAC

Both the presence and quantification of CAC were assessed using a multiple detector computed tomography scan (320 detectors, Aquilion One, Toshiba, Japan). According to standard protocol, images were performed with volumetric acquisition in one heartbeat, triggered at 75% of the cardiac cycle, with 0.5 mm collimation and reconstructed with 3.0 mm slice thickness. All images were analyzed using an off-line workstation (Vitrea, Vital Images, USA) by a blinded and experienced radiologist.

### Biochemical assays

Venous blood was drawn from the arm of the patients. Blood samples were processed, and biochemical and metabolic laboratory profile (ionic calcium, parathormone, vitamin D, creatinine, glycated hemoglobin, total cholesterol, high-density lipoprotein, low-density lipoprotein, triglycerides, apolipoprotein A1 and B) were performed. The following osteogenic and inflammatory proteins were quantified: OPG, fetuin-A, MGP, RANKL, hs-CRP tumor necrosis factor (TNF)-α, transforming growth factor (TGF)-β1, and oxLDL.

Blood samples were processed for osteogenic and inflammatory proteins analysis. The plasma samples were stored at -80°C and assessed simultaneously in order to avoid variation of assay conditions. The quantification of hs-CRP was performed by immunoturbidimetry in the VITROS® 5600 analyzer (Ortho Clinical Diagnostics, USA) with CRP turbiquest plus reagents (Labtest, Brazil). The detection limit of the assay was 0.10 mg/L, and the upper reference limit assigned by the manufacturer was 90.0 mg/L. All other proteins were measured by capture ELISA using commercial duoset sandwich kits (R&D Systems Inc., Minneapolis, Minnesota) to OPG protein, commercial quantikine sandwich kit (R&D Systems Inc., Minneapolis, Minnesota) to fetuin-A, TNF-α, and TGF-β1, and commercial sandwich kit (Cloud-Clone Corp., Houston, USA) to MGP, RANKL, and oxLDL protein. Capture antibody pre-coated 96 wells plates were used in almost all assays. The exception was OPG assay, in which plates were prepared with capture antibody and incubated overnight at room temperature until use. ELISA was performed according to the manufacturer’s protocols. For validation, we did standard curves for all markers according to the manufacturer's instruction, in duplicates, and the reading values of the curves were 99% or higher confidence. Protein concentrations were determined with reference to a standard curve of serial 2-fold dilutions of recombinant proteins. Detection was performed with avidin-horseradish peroxidase (Avidin-HRP) conjugated with tetramethylbenzidine following the manufacturer’s procedure. Absorbance values were measured at 450 nm using a micro-ELISA reader (SpectraMax i3, Molecular Devices). Protein concentrations of test samples were obtained by interpolation from the standard curves. The assays were sensitive in the following concentration ranges: from 62.5 to 4000 pg/mL for OPG; from 7.8 to 250 ng/mL for fetuin-A; from 15.6 to 1000 pg/mL for TNF-α; from 31.3 to 2000-pg/mL for TGF-β1; from 0 to 40 ng/mL for MGP; from 0.156 to 10 ng/mL for RANKL; from 62.5 to 8000 pg/mL for oxLDL.

### Statistical analysis

We estimated our sample size based on the results of a previous study by Crisafulli A et al, which compared levels of plasma OPG among patients with acute MI, patients with stable coronary artery disease and healthy controls, 162 subjects were included [27]. Based on that study, a sample size of 19 patients per group would enable our study to have a power of 90%, with 2-tailed type I error of 0.05, to detect a difference of 1.77 pmol/L in OPG plasma levels between groups. Therefore, we assumed that our sample size (n = 170 patients) would be appropriate to compare OPG serum levels between groups, as well as the other biomarkers.

Continuous variables were expressed as mean ± standard deviation (SD) or medians with interquartile range. Categorical variables were described as absolute and relative frequencies. In CAC patients, clinical profile, metabolic profile, and plasma osteogenic and inflammatory proteins were assessed and compared with control individuals. The -square test and likelihood ratio test were used for categorical variable associations. The Mann-Whitney test and Student t-test were performed for quantitative data comparisons. The adjusted association between plasma biomarkers and CAC was tested by multivariate analysis. Variables included were selected by statistical criteria (univariate p<0.05) and also by clinical relevance, avoiding overfitting the model. Separated full models were performed for the logarithmic transformation of each plasma biomarker, adjusted for age, sex, hypertension, diabetes, treatment with statins and LDL-cholesterol levels, using multivariate logistic regression analysis. In addition, we tested the correlation between all OPs and inflammatory proteins by the Spearman's correlation test.

In patients with MI, metabolic parameters and plasma biomarkers levels were compared between the acute phase and 1–2 months post-MI, by the Wilcoxon signed-ranks test, and use of medication was compared by McNemar's test. Additionally, baseline clinical parameters and metabolic parameters were compared between the acute phase of MI *vs*. control individuals by the chi-square test, the Fisher test or likelihood ratio test. The logarithmic transformed values of inflammatory and osteogenic biomarkers were compared between patients in acute phase of MI *vs*. control individuals by multivariate analysis using generalized linear model with normal distribution and logarithmic link function, adjusted for age, sex, smoking, hypertension, diabetes, total cholesterol levels, parathormone levels and glycated hemoglobin levels.

Missing data was less than 5% and was handled by complete case analysis. All statistical tests were two-sided, and the criterion for statistical significance was *P*<0.05. All statistical analyses were performed using IBM-SPSS for Windows version 20.0.

## Results

### Stable patients with CAC

Compared to controls (60% male, 53 ± 10 years), CAC patients (83% male, 63 ± 10 years, median CAC [25–75 percentile] of 527.5 [255; 1269] AU) were older, predominantly men, had higher rates of hypertension and diabetes, were more frequently treated with aspirin, ACE inhibitors/ angiotensin II receptor blockers, metformin and statins, and had lower levels of plasma LDL-cholesterol and total cholesterol (Table 1).

**Table 1:** Clinical characteristics and metabolic parameters in patients with stable coronary artery calcification (CAC ≥ 100 AU) and patients with acute myocardial infarction, compared to control patients.

Characteristics	Controls (n = 30)	Pts. with CAC (n = 100)	p*	Pts. with acute MI (n = 40)	p**
Male (%)	18 (60)	83 (83)	**0.008**	35 (79.5)	0.067
Mean age, in years	52.7 (9.4)	63.2 (9.6)	**<0.001**	59.5 (9)	**0.003**
Body mass index, in kg/m2	26.8 (4.7)	28 (4)	0.164	28.4 (4)	0.116
**Medications**					
ACEi/ARBs (%)	7 (23.3)	53 (53)	**0.004**	27 (67.5)	**<0.001**
Aspirin (%)	4 (13.3)	45 (45)	**0.002**	40 (100)	**<0.001**
Metformin (%)	2 (6.7)	28 (28)	**0.015**	9 (22.5)	0.100
Statin (%)	10 (33.3)	76 (76)	**<0.001**	40 (100)	**<0.001**
Ezetimibe (%)	2 (6.7)	11 (11)	0.731	3 (7.5)	>0.999
Fibrate (%)	0 (0)	3 (3)	>0.999	1 (2.5)	>0.999
ADP antagonist (%)	0 (0)	3 (3)	>0.999	40 (100)	**<0.001**
Calcium supplement (%)	0 (0)	2 (2)	>0.999	NA	
Vitamin D supplement (%)	1 (3.3)	9 (9)	0.452	NA	
Alendronate (%)	0 (0)	2 (2)	>0.999	NA	
**Cardiovascular Risk Factors**					
Current smoker (%)	3 (10)	9 (9)		16 (36.4)	**0.011**
Hipertension (%)	10 (33.3)	64 (64)	**0.003**	28 (63.6)	**0.01**
Diabetes (%)	2 (6.7)	27 (27)	**0.019**	12 (27.3)	**0.026**
Calcium score, ui	0	527.5 (255; 1269)	**<0.001**	NA	
**Laboratory tests**					
Ionized calcium, mg/dl	4.92 (4.8; 5.04)	4.8 (4.64; 4.88)	**<0.001**	NA	
Creatinine, mg/dL	0.86 (0.7; 1)	0.9 (0.77; 1)	0.274	0.9 (0.8; 1.1)	0.101
Apolipoprotein A-1, mg/dL	136 (23.2)	131.6 (25.1)	0.391	118.8 (19.4)	**0.001**
Apolipoprotein B, mg/dL	90 (22.5)	81.3 (28.6)	0.130	84.6 (23.4)	0.176
Total cholesterol, mg/dL	181.6 (40.7)	157.8 (39.2)	**0.004**	159.9 (38.9)	**0.015**
HDL-cholesterol, mg/dL	47.9 (14.7)	45.4 (18.6)	0.495	39 (12.4)	**0.009**
LDL-cholesterol, mg/dL	104.5 (34.8)	88.9 (32.9)	**0.027**	92 (31.1)	0.105
Triglycerides, mg/dL	140.5 (66.8)	145.7 (89.6)	0.771	150.3 (100.9)	0.915
Glycated hemoglobin, %	5.61 (0.39)	6.45 (5.42)	0.401	6.1 (1)	**0.047**
Parathormone, pg/mL	46.3 (37.5; 57.6)	41.5 (34.5; 53.7)	0.206	41.6 (15.2)	**0.037**
Vitamin D, ng/ml	26 (22.8; 39.8)	30 (24; 36)	0.468	25.7 (10)	0.138

Data are expressed as mean ± standard deviation (SD), median (25th–75th percentile), or number (%).Pts, patients; ACEi, angiotensin converting enzyme inhibitor; ARB, angiotensin II receptor blocker; ADP, adenosine diphosphate receptor; HDL, high density lipoprotein; LDL, low density lipoprotein; MI, myocardial infarction.

*Comparison between CAC patients and control patients

**Comparison between acute myocardial infarction patients and control patients.

Significant p values are in boldface.

**Fig 1:**
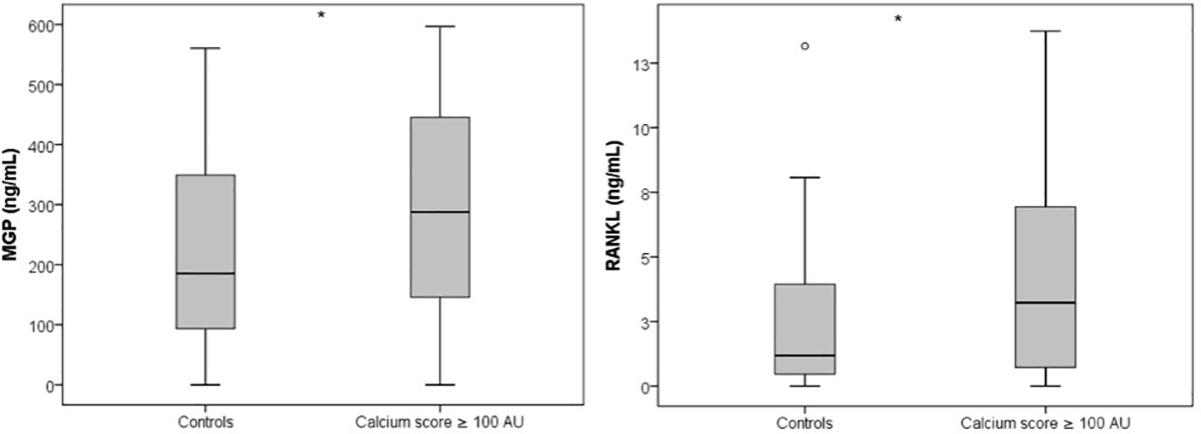
Plasma levels of MGP and RANKL in stable patients with CAC ≥ 100 AU and in control patients (CAC = 0 AU). MGP, matrix Gla protein; RANKL, receptor activator of nuclear factor kappa-B ligand; CAC, coronary artery calcification; AU, Agatston units. *Adjusted p-values of 0.020 and 0.035, respectively for MGP and RANKL.

Among the osteogenic and inflammatory proteins, both MGP and Fetuin-A levels were significantly higher in CAC patients, compared to control individuals (respectively, median [25–75 percentile] of 287.6 [145–447.6] *vs*. 185.4 [91.3–351.8] ng/ml, p = 0.041 and 811.4 [603.7–1085] *vs* 644.8 [457.7; 888.7] μg/ml, p = 0.022; Table A in S1 Table). After adjusted analysis, higher plasma levels of both MGP and RANKL were associated with CAC (Table 2, Fig 1). In this group of patients, among the biomarkers, TGF-β1 plasma levels correlated positively with OPG (r = 0.52; p < 0.001). No other biomarkers were correlated.

**Table 2:** Multivariate analysis on the association between plasma biomarkers and CAC (final model).

Biomarkers	OR (CI: 95%)	p
**Univariate analisys**		
Male (%)	3.26 (1.33; 7.99)	**0.008**
Mean age, in years	1.12 (1.06; 1.18)	**<0.001**
Statin (%)	6.33 (2.61; 15.38)	**<0.001**
Hipertension (%)	3.56 (1.50; 8.42)	**0.003**
Diabetes (%)	5.18 (1.15; 23.23)	**0.019**
LDL-cholesterol, mg/dL	0.99 (0.98; 0.999)	**0.027**
Osteoprotegerin	0.94 (0.56; 1.58)	0.824
RANKL	1.24 (0.89; 1.72)	0.207
Fetuin A	2.36 (0.80; 6.92)	0.119
MGP	1.87 (1.06; 3.30)	**0.030**
hs-CRP	0.90 (0.38; 2.10)	0.801
oxLDL	1.19 (0.99; 1.44)	0.071
TNF-α	0.88 (0.71; 1.09)	0.231
TGF-β	0.95 (0.64; 1.42)	0.801
**Multivariate analisys**		
Osteoprotegerin	1.18 (0.51; 2.72)	0.706
RANKL	1.75 (1.04; 2.94)	**0.035**
Fetuin A	1.79 (0.41; 7.94)	0.441
MGP	3.12 (1.20; 8.11)	**0.020**
hs-CRP	1.25 (0.34; 4.63)	0.740
oxLDL	1.27 (0.95; 1.71)	0.111
TNF-α	0.82 (0.60; 1.14)	0.241
TGF-β	0.70 (0.40; 1.24)	0.222

Adjusted for age, sex, hypertension, diabetes, treatment with statins and LDL-cholesterol levels. OR, odds ratio; RANKL, receptor activator of nuclear factor kappa-B ligand; mgp, matrix Gla protein; CRP, C-reactive protein; oxLDL, oxidized low density lipoprotein; TNF, tumor necrosis factor-α; TGF, tumor growth factor. Significant p values are in boldface.

### Patients with myocardial infarction

Forty patients with acute MI were enrolled (80% male, 60±9 years, median [25–75 percentile] peak troponin of 14.3 [2.7–29.1] μg/ml). All patients with ST-elevation MI (59%) were successfully treated with primary angioplasty. Non ST-elevation MI patients were managed with early invasive strategy and performed percutaneous transluminal coronary angioplasty if indicated.

Compared to control patients, patients with acute MI were older, had higher rates of hypertension, diabetes and smoking, were more frequently treated with aspirin, ACE inhibitors/ angiotensin II receptor blockers and statins, and had lower levels of plasma LDL-cholesterol, HDL-cholesterol, total cholesterol, apolipoprotein-A, parathormone, and slightly higher levels of glycated hemoglobin (Table 1). After adjusted analysis, patients with acute MI had higher levels of hs-CRP, oxLDL and OPG compared to control patients (Table 3).

**Table 3:** Plasma biomarkers in patients with acute myocardial infarction and control patients.

Biomarkers	Controls (n = 30)	Pts. with acute MI (n = 40)	p	p*
**Osteoprotegerin, pg/ml**	102 (0; 430.2)	380.5 (173.4; 590)	**0.008**	**0.008**
**RANKL, ng/ml**	1.2 (0.4; 4)	3.5 (0; 6.2)	0.505	0.358
**Fetuin A, μg/ml**	644.8 (457.7; 888.7)	760.9 (545.5; 955.9)	0.296	0.951
**MGP, ng/ml**	185.4 (91.3; 351.8)	278.2 (161.7; 352.7)	0.214	0.192
**hs-CRP, mg/L**	0 (0; 2.9)	19.1 (8.8; 36.5)	**<0.001**	**0.026**
**oxLDL, ng/ml**	44.7 (6; 854)	533.7 (24.3; 988.1)	0.282	**0.031**
**TNF-α, pg/ml**	37.1 (7.9; 627.7)	19.9 (7.9; 252.9)	0.465	0.228
**TGF-β1, pg/ml**	570.8 (0; 708.7)	510.7 (0.2; 797.1)	0.685	0.495

Data are expressed as median (25th–75th percentile). RANKL, receptor activator of nuclear factor kappa-B ligand; MGP, matrix Gla protein; CRP, C-reactive protein; oxLDL, oxidized low density lipoprotein; TNF, tumor necrosis factor-α; TGF, tumor growth factor.

* Multivariate analysis using generalized linear model with normal distribution and logarithmic link function, adjusted for age, sex, smoking, hypertension, diabetes, total cholesterol, parathormone and glycated hemoglobin. Significant p values are in boldface.

In the subset of patients with MI, the use of cardiovascular medication was similar in both acute phase and 1–2 months post-MI. Among the 40 patients, 29 (72.5%) were naïve from statins before hospital admission. All patients were treated with statins both in the acute phase and 1–2 months post-MI. High-intensity statin (i.e. atorvastatin 40–80 mg, rosuvastatin 20–40 mg or ezetimibe/simvastatin 10/40 mg) treatment rates were also similar in the acute phase and 1–2 months post-MI (57.5% *vs*. 62.5%; p = 0.727. Table B in S1 Table). Compared to the acute phase of MI, plasma levels of LDL-cholesterol were markedly decreased and those of HDL-cholesterol were slightly increased 1–2 months post-MI (Table B in S1 Table). Looking at the plasma inflammatory and osteogenic proteins, we found that by 1–2 months post-MI the hs-CRP levels were decreased, whereas both TGF-β1 and MGP levels were increased in comparison to those of the acute phase of MI (Table 4).

**Table 4:** Plasma biomarkers in patients with myocardial infarction, in the acute phase and 1–2 months post-MI.

Pts. with MI (n = 40)	Acute phase MI	1–2 months post-MI	p
**Biomarkers**			** **
**Osteoprotegerin, pg/ml**	380.5 (173.4; 590)	322.3 (149.3; 664.7)	0.896
**RANKL, ng/ml**	3.5 (0; 6.2)	3.7 (1.3; 8.1)	0.766
**Fetuín A, μg/ml**	760.9 (545.5; 955.9)	762.5 (537.1; 1057)	0.694
**MGP, ng/ml**	278.2 (161.7; 352.7)	342 (257.4; 433.6)	**0.014**
**hs-CRP, mg/L**	19.1 (8.8; 36.5)	0 (0; 0)	**<0.001**
**oxLDL, ng/ml**	533.7 (24.3; 988.1)	768.3 (445.5; 1061)	0.201
**TNF-α, pg/ml**	19.9 (7.9; 252.9)	37.8 (13.3; 618.4)	0.072
**TGF-β1, pg/ml**	510.7 (0.2; 797.1)	711.3 (427.2; 904.9)	**0.014**

Data are expressed as median (25th–75th percentile). Pts, patients; RANKL, receptor activator of nuclear factor kappa-B ligand; MGP, matrix Gla protein; CRP, C-reactive protein; oxLDL, oxidized low density lipoprotein; TNF, tumor necrosis factor-α; TGF, tumor growth factor. Significant p values are in boldface.

In the acute MI phase, correlations within the plasma levels of biomarkers were also found. TGF-β1 correlated positively with OPG (r = 0.54; p = 0.001) and also with RANKL (r = 0.65; p < 0.001) levels. oxLDL plasma levels were positively correlated with RANKL (r = 0.56; p < 0.001). Acute phase plasma osteogenic and inflammatory protein levels were not correlated to the levels of these biomarkers 1–2 months post-MI.

## Discussion

### Association between circulating MGP and RANKL with CAC

The present study reports that, among several plasma inflammatory or osteogenic proteins tested, higher MGP and RANKL levels were the only biomarkers associated with CAC in our cohort of stable ambulatory patients. MGP is a Vitamin K-dependent protein secreted by vascular smooth muscle cells, endothelial cells and fibroblasts of the vascular wall.[5] MGP inhibits ectopic calcification by various mechanisms e.g. by directly decreasing hydroxyapatite aggregation, by preventing calcium accrual in elastic lamina (a nucleation site for calcium binding that initiates ectopic mineralization), and by inhibiting bone morphogenetic protein activity, an important cellular signaling mechanism towards vascular calcification.[28]

Despite experimental studies show that MGP inhibits ectopic calcification, few clinical studies that tested the association between circulating MGP and CAC or CAD found conflicting results. A small cohort study suggested that elevated plasma MGP levels were positively associated with CAD [11], while other larger clinical studies found no association between plasma MGP and CAC. Additionally, one study showed an inverse association between higher plasma MGP and CAC.[12–14] The cardiovascular prognostic value of plasma MGP is also unclear. In patients with stable vascular disease, higher plasma desphospho-uncarboxylated MGP levels were associated with adverse cardiovascular outcomes. [29] In contrast, other authors did not find this association when examining a larger cohort of patients. Still another investigation on stable CAD patients found an inverse association between higher uncarboxylated MGP levels and the risk of cardiovascular adverse events and death. [30, 31] Finally, a study in a cohort of diabetic patients concluded that higher levels of uncarboxylated MGP, but not carboxylated MGP, were positively associated with long-term cardiovascular risk. [32] Therefore, because of the conflicting findings from the several studies, there is no consensus on the prognostic value of MGP as a biomarker in cardiovascular clinical settings. The discrepancies may relate to the diversity of the enrolled populations in those studies and/or, perhaps more importantly, to the fact that distinct MGP-quantifying methodologies may detect MGP subtypes having different degrees of phosphorylation and carboxylation. Interestingly, despite the anti-osteogenic properties of MGP, high concentrations were detected in human calcified arteries [33] as coincidently demonstrated in animal models of diabetes and vascular calcification.[34] Therefore, taken together with previous evidence our findings suggest that MGP increase in patients with CAC could possibly reflect a counter-regulatory effect of vascular calcification.

The OPG/RANKL/RANK axis regulates osteoclastic activation and bone reabsorption. The imbalance among the components of this axis increases bone reabsorption and vascular calcification. The binding of RANKL to RANK on the surface of osteoclasts or dendritic cells triggers the NF-κB pathway leading to osteoclast differentiation [18]. OPG, which is expressed in bone and vascular tissue (vascular smooth muscle cells and endothelial cells) [35] is a soluble decoy receptor, competing for RANKL; by binding to RANK, OPG inhibits RANKL/RANK interaction and activity. OPG-genetically-deficient mice have osteoporosis and intense vascular calcification.[36]

The association between circulating RANKL and CAC was scarcely investigated in clinical studies. Small studies on specific populations did not find association between circulating RANKL levels and CAC. [37, 38] A large prospective study reported that baseline levels of circulating OPG, but not RANKL, were positively associated with a higher risk of future cardiovascular events. [39]. Interestingly, one clinical trial has investigated the effect of denosumab on RANKL axis inhibition, reporting that it failed to attenuate aortic calcification progression. [40]

Our results suggest that the RANKL/OPG axis may be clinically relevant for CAC progression, and unveil a potential value of circulating RANKL and MGP, as surrogate biomarkers of CAC, although the present findings need to be further tested in larger cohort studies.

### Osteogenic modulation in patients with MI

Our findings suggest that besides the inflammatory response, osteogenic modulation was activated after MI. We observed, in the acute phase of MI, elevation in plasma OPG, hs-CRP and oxLDL levels, compared to controls. After 1–2 months post-MI, compared to the acute phase, hs-CRP levels were decreased, while OPG and oxLDL levels were maintained, and TGF-β1 and MGP levels were further increased. Additionally, the inflammatory proteins TGF-β1 and oxLDL levels correlated with elevated OPs plasma levels (OPG and RANKL) in the acute phase of MI. Of note, hs-CRP values 1–2 months post MI were lower than expected. This finding could be related to the fact that all patients were treated with statins (~60% with high-intensity statin treatment), with a marked LDL cholesterol reduction during follow-up. On the other hand, despite hs-CRP and LDL cholesterol reduction, higher levels of oxLDL observed in the acute phase were maintained 1–2 months post-MI, finding that may indicate the presence of persistent oxidative stress after MI.

Previous clinical studies have shown that the elevation of both OPG and oxLDL plasma levels in patients with recent MI was associated with the severity of MI, with heart failure, and with long-term adverse outcomes.[23, 25]. In accordance with our results, Shetelig C et al. demonstrated that circulating OPG levels were increased in the acute phase of ST-elevation MI patients and were only slightly decreased after 4 months, suggesting that OPG levels may remain increased after acute and subacute MI. [25]

On the other hand, MGP plasma levels were seldom investigated in patients with acute MI. A study on a small number of patients failed to find differences on the levels of plasma MGP between patients with acute MI and patients with stable CAD.[41]. As we found elevated plasma MGP levels by 1–2 months after MI, the underlying mechanisms are, at this moment, hypothetical. Cross-talk between MGP and inflammatory pathways is a plausible hypothesis because in experimental studies, inflammatory proteins regulate osteogenic proteins such as RANKL, OPG and bone morphogenic proteins. [5]

TGF-β1 is synthesized by inflammatory cells and platelets and exerts such anti-atherogenic functions as inhibition of smooth muscle cells proliferation as well as of leukocyte adhesion, neutrophil migration, foam cell synthesis and apoptosis.[42–44] On the other hand, TGF-β1stimulates vascular osteogenesis by intracellular signaling via the transcription activators SMADS to initiate osteogenic programming by increasing RUNX-2 (runt-related transcription factor-2) expression.[45] In patients with acute MI, TGF-β1 exerts pleiotropic anti-inflammatory effects on monocytes, lymphocytes and macrophages, but also induces pathological ventricular remodeling. It also stimulates collagen and fibronectin synthesis, and angiotensin I and II receptors.[46] In addition, TGF-β1 may influence post-MI thrombotic risk because it stimulates the synthesis of tissue plasminogen activator inhibitor.[47] A clinical study on a small patients cohort found increased TGF-β plasma levels up to one month post-MI.[48] A larger cohort study found that plasma TGF-β1 levels were higher in patients with acute MI, compared to patients with stable CAD and healthy controls.[49] Still other authors found that the expression of TGF-β1 in activated platelets of patients with acute coronary syndromes was higher than in patients with stable CAD, a finding associated with adverse outcomes. [50] In our study, we did not find an increase in TGF-β1 levels in MI acute phase, but we follow the same patients and demonstrate a further increase in TGF-β1 levels 1–2 months post-MI, finding that may be related to myocardial remodeling and healing, generally observed during the first month post-MI.

Summarizing our findings in MI patients, we observed an up-regulation of the expression of OPG and MGP, respectively during the acute phase and 1–2 months post MI, together with acute phase inflammation and residual inflammatory and oxidative activation 1–2 months post MI (i.e. high levels of oxLDL and TGF-β1). Therefore, taken together with experimental evidence on the interrelation between inflammation and OPs regulation, our findings suggest that the regulation of OPG and MGP is affected by atherothrombotic inflammation. The mechanisms behind the regulation of these OPs expression after MI remain unknown and should be investigated in further translational studies. Nevertheless, our results raise the hypothesis that atherothrombotic inflammation could trigger upregulation of the expression of selected OPs that possibly counter-regulate vascular calcification, such as OPG and MGP, as a possible explanation of why clinical studies failed to show a positive association between circulating inflammatory proteins and CAC. [21, 22]

Moreover, studies that measured OPs during acute MI may have underestimated the inflammatory effects of MI on osteogenic modulation, considering that we found further increase in the levels of circulating osteogenic and inflammatory proteins up to 1–2 months after MI. In addition, our findings suggest that the prognostic role of these biomarkers should be assessed in the acute phase and 1–2 months post-MI.

### Limitations

Our study has some important limitations. The relatively small sample size of our population may have been insufficient to detect differences of biomarkers between groups, or between acute phase and 1–2 months post-MI phase, considering its asymmetric distribution (e.g. RANKL and MGP circulating levels were associated with CAC, but were not significantly increased in MI patients, compared to controls). In addition, control patients were significantly younger than patients with CAC, and also than MI patients. We approached this limitation by including age in all adjusted models. Another limitation of our study methodology is that we did not assess functional activity of MGP, determined by post-translational modification, such as carboxylation of glutamic residues and/or phosphorylation of serine, because no antibodies or ELISA against the modified protein is commercially available. Of note, this clinical study was not designed to assess the mechanisms causing the increase of OPs levels either in stable individuals with CAC or in MI patients. The mechanistic effect of post-MI inflammatory activation in the regulation of OPs expression was not investigated. Thus, future studies should further investigate the mechanisms of regulation of OPs in patients with coronary atherosclerosis, and their relation to CAC progression. In addition, larger cohort clinical studies should confirm the role of circulating OPs as surrogate biomarkers of CAC, and as prognostic markers after MI.

## Conclusions

In conclusion, we report that higher circulating levels of MGP and RANKL are independently associated with CAC in stable patients. These findings highlight the potential value of these proteins as modulators and markers of CAC. In addition, the increase in circulating OPG and MGP, as well as of inflammatory proteins levels after MI, suggest that the regulation of these OPs is affected by increased atherothrombotic inflammation. Understanding the mechanisms underlying CAC modulation by these proteins can pave the way to innovative medical therapy for atherosclerotic calcification.

## Supporting information

S1 Dataset Stable patients with CAC and controls. (XLS)

S2 Dataset Myocardial infarction group. (XLS)

S1 Clinical File PODAC English version. (DOC)

S2 Clinical File PODAC Portuguese version. (DOC)

S1 Table PODAC. (DOCX)

## Abbreviations

MI: Myocardial infarction
CAC: Coronary artery calcification
CAD: Coronary artery disease
AU: Agatston units
TGF-β1: Tumor growth factor
OPG: Osteoprotegerin
MGP: Matrix Gla protein
RANKL: Receptor activator of nuclear factor kappa-B ligand
Ox: oxidized
TNF: tumor necrosis factor-α
TGF: transforming growth factor
HDL: high density lipoprotein
LDL: low density lipoprotein
