# Supplementary material for: Circulating osteogenic proteins are associated with coronary artery calcification and increase after myocardial infarction
Source: PLoS One. 2018 Aug 23;13(8):e0202738. doi: 10.1371/journal.pone.0202738 (PMC6107213; doi:10.1371/journal.pone.0202738)
Supplement: S1 Clinical File — (DOC) [file pone.0202738.s003.doc]

| Estudo PODAC (****Einstein/SGPP 1666-12*;***** FAPESP 2013/06799-0)“Evaluation of osteogenic modulating proteins and inflammatory profile of patients with acute myocardial infarction and stable individuals with coronary artery calcification "Principal investigator: Antonio Eduardo Pereira Pesaro |
| --- |

IDENTIFICATION

| Nº study: | Nº file Einstein: Inclusion date: |
| --- | --- |

| Name: |
| --- |
| CPF:  Email: |
| Adress: |
| Tel: Celular: Office: |

- PATIENT IS NOT PARTICIPATING IN ANOTHER STUDY ()
- PATIENT SIGNED CONSENT ()
- GROUP INCLUSION?
- STABLE WITH CAC≥100
- STABLE WITH CAC = ZERO
- AMI

**Inclusion Criteria**

AMI pts:

1. Acute MI was defined according to international guidelines criteria: typical rise and gradual fall of biochemical markers of myocardial necrosis (troponin or creatine kinase-MB) with at least one of the following: 1) ischemic symptoms, 2) development of pathologic Q waves on the electrocardiogram (ECG), 3) ECG changes indicative of ischemia (ST-segment elevation or depression), or 4) coronary artery intervention (e.g., coronary angioplasty).

2. Age >35 , < 80 y

Stable pts with CAC:

Cardiovascular stable patients submitted to ambulatory elective CT-coronary angiography in the routine of the hospital service and who presented a significant CAC presence (calcium score≥100).

Stable pts with CAC zero:

Cardiovascular stable patients submitted to ambulatory elective CT-coronary angiography in the routine of the hospital service and who presented a CAC zero.

**Exclusion criteria:**

AMI pts:

Previous coronary artery bypass graft surgery, recent (< 3 months) MI, recent (< 3 months) cardiac or non-cardiac surgical procedures, chronic kidney disease (creatinine > 1.5 mg/dL), cancer, chronic obstructive pulmonary disease, current infections, vasculitis, collagen diseases, inflammatory chronic diseases and immunosuppressive treatment.

Stable pts:

Previous MI, previous coronary angioplasty or coronary artery bypass graft surgery, recent (< 3 months) non-cardiac surgical procedures, chronic kidney disease (creatinine > 1.5 mg/dL), cancer, chronic obstructive pulmonary disease, current infections, vasculitis, collagen diseases, chronic inflammatory diseases and immunosuppressive treatment.

**VISIT 1 (SCREENING)**

Date: _______________

DD MMM AAAA

| Demographic data | | | | | | | | | | | | |
| --- | --- | --- | --- | --- | --- | --- | --- | --- | --- | --- | --- | --- |
| Age y: |  |  |  | Sex: | | Fem | |  | Masc | |  |  |
|  | | | | | | | | | | | | |
| Race: | | | | | | | | | | | | |
| Height (m): | | | | |  |  |  |  |  |  |  |  |
|  | | | | | | | | | | | | |
| Weight (Kg): | | | | |  |  |  |  |  |  |  |  |
|  | | | | | | | | | | | | |
| Body mass index (BMI = Weight (kg) / Height2 (M): | | | | |  |  |  |  |  |  |  |  |
|  | | | | | | | | | | | | |

| Smoking | | | | | | | | | |
| --- | --- | --- | --- | --- | --- | --- | --- | --- | --- |
| Current or past smoker? Yes No | | | | | Sim* |  | Não |  |  |
|  | | | | | | | | | |
|  | |  |  |  | | | | | |
| How many cigarettes per day? How many years? How many years did it stop? | | | | | | | | | |
|  | |  |  | | --- | --- | | | | | | | | | |

| Medications | Yes |  | No |  |  |
| --- | --- | --- | --- | --- | --- |

| IECA (enalapril, Ramipril, etc) | yes | name |
| --- | --- | --- |
| ARB/ACEI (Losartan, irbesartan, etc)) |  |  |
| AAS |  |  |
| Sulfonilurieia (glimepirida, etc) |  |  |
| METFORMINE |  |  |
| INSULINE |  |  |
| NITRATES |  |  |
| (ANLODIPINA, etc) |  |  |
| B.B.: PROPANOLOL ( ) ATENOL ( ) |  |  |
| Estatin |  | Dose/start? |
| ADP INHIBITOR (CLOPIDOGREL, ticagrelor, prasugrel)  Calcium Replacement  Vitamin D Replenishment  Alendronate / similar |  |  |

| PREVIOUS MEDICAL HISTORY | | | | | | | | |
| --- | --- | --- | --- | --- | --- | --- | --- | --- |
| Is there any relevant medical history in the following systems? | | | | | | | | |
| Cod. | SYSTEM | YES | No |  | Code | SYSTEM | yes | No |
| 1 | System  Cardiovascular  Respiratory  Hepatobiliary  Gastro-instestinal  Genito-urinary  Endocrine / diabetes  Hematological  Skeletal muscle | | |  | 9 | Cancer  Neurol  Others | | |
| 2 |  |  |  |  | 10 |  |  |  |
| 3 |  |  |  |  |  |  |  |  |
| 4 |  |  |  |  |  |  |  |  |
| 5 |  |  |  |  |  |  |  |  |
| 6 |  |  |  |  |  |  |  |  |
| 7 |  |  |  |  |  |  |  |  |
| 8 |  |  |  |  | 00 |  |  |  |

* If YES for any of the above situations, enter the code for each condition in the boxes below, provide more details (including dates) and whether the condition is currently or potentially active.

|  | | Active Symptom | |
| --- | --- | --- | --- |
| Cód. | Details | yes | no |
|  | Typical precordial pain? |  |  |
|  | Atypical precordial pain? |  |  |

Diagnostic tests

| EKG: | Normal |  | Abnormal |  | ** |
| --- | --- | --- | --- | --- | --- |
| **Description: |  | | | | |

| ECHO: | Normal |  | Abnormal |  | LVEF (%): |
| --- | --- | --- | --- | --- | --- |
| **Description: |  | | | | |

| ANGIOGRAPHY | Normal |  | Abnormal |  | ** |
| --- | --- | --- | --- | --- | --- |
| **Description: |  | | | | |

| CT-CORONARY | Normal |  | Abnormal |  | SCORE CAC |
| --- | --- | --- | --- | --- | --- |
| **Description: |  | | | | |

| STRESS TEST | Normal |  | Abnormal |  | ** |
| --- | --- | --- | --- | --- | --- |
| **Description: |  | | | | |

| Peak troponine |  |  |  |  |  |
| --- | --- | --- | --- | --- | --- |
| BNP |  | | | | |

Signature: Date:

VISIT 2 (MI group) Date:______________
