# Supplementary material for: Circulating osteogenic proteins are associated with coronary artery calcification and increase after myocardial infarction
Source: PLoS One. 2018 Aug 23;13(8):e0202738. doi: 10.1371/journal.pone.0202738 (PMC6107213; doi:10.1371/journal.pone.0202738)
Supplement: S2 Clinical File — (DOC) [file pone.0202738.s004.doc]

| Estudo PODAC (****Einstein/SGPP 1666-12***;*** FAPESP 2013/06799-0)“Avaliação das proteínas moduladoras da osteogênese e do perfil inflamatório de pacientes com infarto agudo do miocárdio e indivíduos estáveis com calcificação arterial coronária”Investigador Principal: Antonio Eduardo Pereira Pesaro |
| --- |

ETIQUETA

| Nº no Estudo: | Nº do prontuário Einstein: Data de Inclusão: |
| --- | --- |

| Nome: |
| --- |
| CPF:  Email: |
| Endereço: |
| Telefone domicílio: Celular: Trabalho: |

- PACIENTE NÃO ESTÁ PARTICIPANDO DE OUTRO ESTUDO ( )
- PACIENTE ASSINOU TERMO DE CONSENTIMENTO ( )
- GRUPO INCLUSÃO?
- ESTÁVEL COM CAC≥100
- ESTÁVEL COM CAC=ZERO
- IAM

**Critérios de inclusão:**

**Grupo de pacientes com IAM:**

1. Pacientes com critérios diagnósticos de IAM (aumento característico e diminuição gradual da troponina ou aumento e diminuição mais rápidos para creatinaquinase CK fração MB, com pelo menos um dos seguintes critérios: a) sintomas isquêmicos; b) alterações eletrocardiográficas indicativas de isquemia; c) desenvolvimento de ondas Q patológicas no eletrocardiograma; ou d) evidência, em exames de imagem, de perda de viabilidade miocárdica ou contratilidade segmentar anormal).?

- **SIM**

2. Idade >35 anos e < 80 anos

- **SIM**

**Grupo de pacientes estáveis com CAC:**

1. Pacientes estáveis do ponto de vista cardiovascular, submetidos à angio-tomografia de coronária eletiva ambulatorial na rotina do serviço hospitalar e que apresentaram presença de CAC significativa (score de cálcio≥100).

- **SIM**

**Grupo de pacientes estáveis controle CAC zero:**

1. Pacientes estáveis do ponto de vista cardiovascular, submetidos à angio-tomografia de coronária eletiva na rotina do serviço hospitalar e que apresentam score de cálcio=zero.

- **SIM**

**Critérios de exclusão:**

Grupo IAM: Pacientes com IAM associado a procedimento percutâneo ou cirurgia de revascularização miocárdica; pacientes em Killip > 2; pacientes com Insuficiência renal (Creatinina > 1.5 mg/dL), neoplasias, DPOC, infecções vigentes, doenças do tecido conectivo, doenças inflamatórias crônicas ou tratamento com imunossupressores.

- **Não há**

Grupo de pacientes estáveis ambulatoriais: pacientes com IAM prévio, stent prévio, revascularização do miocárdio prévia, cirurgias de grande porte recentes (<3 meses), insuficiência renal (Creatinina > 1.5 mg/dL), neoplasias, DPOC, infecções vigentes, doenças do tecido conectivo, doenças inflamatórias crônicas ou tratamento com imunossupressores.

- **Não há**

**VISITA 1 (SCREENING)**

**Data: _______________**

**DD MMM AAAA**

| DADOS DEMOGRÁFICOS | | | | | | | | | | | | |
| --- | --- | --- | --- | --- | --- | --- | --- | --- | --- | --- | --- | --- |
| Idade (anos): |  |  |  | Sexo: | | Feminino | |  | Masculino | |  |  |
|  | | | | | | | | | | | | |
| Raça: | | | | | | | | | | | | |
| Altura (m): | | | | |  |  |  |  |  |  |  |  |
|  | | | | | | | | | | | | |
| Peso (Kg): | | | | |  |  |  |  |  |  |  |  |
|  | | | | | | | | | | | | |
| Índice de massa corpórea (IMC = Peso (kg)/Altura^2^ (M): | | | | |  |  |  |  |  |  |  |  |
|  | | | | | | | | | | | | |

| Tabagismo | | | | | | | | | |
| --- | --- | --- | --- | --- | --- | --- | --- | --- | --- |
| Fumante atual ou passado? | | | | | Sim* |  | Não |  |  |
|  | | | | | | | | | |
| *****Quantos cigarros por dia? | |  |  |  | | | | | |
|  | | | | | | | | | |
| Há quantos anos?  Parou há quanto anos? | |  |  | | --- | --- | | | | | | | | | |

| **IECA (enalapril, Ramipril, etc)** | **SIM** | **Nome** |
| --- | --- | --- |
| **BRA (Losartan, irbesartan, etc))** |  |  |
| **AAS** |  |  |
| **Sulfonilurieia (glimepirida, etc)** |  |  |
| **METFORMINA** |  |  |
| **INSULINA** |  |  |
| **NITRATOS** |  |  |
| **Bloquedor de canais de calico (ANLODIPINA, etc)** |  |  |
| **B.B.: PROPANOLOL ( ) ATENOL ( )** |  |  |
| **Estatina** |  | **Especificar dose** |
| **Inibidor de ADP (CLOPIDOGREL, ticagrelor, prasugrel)**  **Reposição de Cálcio**  **Reposição de Vitamina D**  **Alendronato/similares** |  |  |

| **Medicações em uso?** | Sim |  | Não |  |  |
| --- | --- | --- | --- | --- | --- |

| HISTÓRIA MÉDICA PRÉVIA | | | | | | | | |
| --- | --- | --- | --- | --- | --- | --- | --- | --- |
| **Há alguma história médica relevante nos seguintes sistemas?** | | | | | | | | |
| Cod. | Sistema | *Sim | Não |  | Code | Sistema | *Sim | Não |
| 1 | Cardiovascular |  |  |  | 9 | Neoplasia |  |  |
| 2 | Respiratório |  |  |  | 10 | Neurológico |  |  |
| 3 | Hepato-biliar |  |  |  |  |  |  |  |
| 4 | Gastro-instestinal |  |  |  |  |  |  |  |
| 5 | Genito-urinário |  |  |  |  |  |  |  |
| 6 | Endócrino/diabetes |  |  |  |  |  |  |  |
| 7 | Hematológico |  |  |  |  |  |  |  |
| 8 | Músculo-esquelético |  |  |  | 00 | Outros |  |  |

* Se SIM para alguma das situações acima, digite o código para cada condição nas caixas abaixo, fornecer mais detalhes (incluindo datas) e se a condição é atualmente ou potencialmente ativa.

|  | | Condição Ativa? | |
| --- | --- | --- | --- |
| **Cód.** | **Detalhes (incluir datas)** | **Sim** | **Não** |
|  | Dor precordial típica? |  |  |
|  | Dor precordial atípica? |  |  |

Exames Subsidiários

| **ECG:** | Normal |  | Anormal |  | ****** |
| --- | --- | --- | --- | --- | --- |
| **Descrição: |  | | | | |

| **ECOCARDIOGRAMA:** | Normal |  | Anormal |  | **Menor FEVE (%):** |
| --- | --- | --- | --- | --- | --- |
| **Descrição da anormalidade: |  | | | | |

| **CINEANGIOCOR.** | Normal |  | Anormal |  | ****** |
| --- | --- | --- | --- | --- | --- |
| **Descrição: |  | | | | |

| **TOMOGRAFIA COR.** | Normal |  | Anormal |  | **ESCORE Ca?** |
| --- | --- | --- | --- | --- | --- |
| **Descrição: |  | | | | |

| **TESTE ISQUÊMIA:** | Normal |  | Anormal |  | ****** |
| --- | --- | --- | --- | --- | --- |
| **Descrição: |  | | | | |

| **Pico de Troponina:** |  |  |  |  |  |
| --- | --- | --- | --- | --- | --- |
| BNP mais alto |  | | | | |

Assinatura: Data:

**VISITA 2 (GRUPO IAM) Data:______________**
