# Supplementary material for: Circulating osteogenic proteins are associated with coronary artery calcification and increase after myocardial infarction
Source: PLoS One. 2018 Aug 23;13(8):e0202738. doi: 10.1371/journal.pone.0202738 (PMC6107213; doi:10.1371/journal.pone.0202738)
Supplement: S1 Table — (DOCX) [file pone.0202738.s005.docx]

**Supporting Tables**

Table A. Plasma biomarker levels in patients with stable CAC and in control patients.

| **Biomarkers** | **Controls (n = 30)** | **Pts. with CAC ≥ 100 AU (n = 100)** | **p** |
| --- | --- | --- | --- |
| **Osteoprotegerin, pg/ml** | 102 (0; 430.2) | 165.8 (1.4; 507.7) | 0.401 |
| **RANKL, ng/ml** | 1.2 (0.4; 4) | 3.2 (0.7; 7) | 0.134 |
| **Fetuín A, µg/ml** | 644.8 (457.7; 888.7) | 811.4 (603.7; 1085) | **0.022** |
| **MGP, ng/ml** | 185.4 (91.3; 351.8) | 287.6 (145; 447.6) | **0.041** |
| **hs-CRP, mg/L** | 0 (0; 2.9) | 0 (0; 2.7) | 0.829 |
| **oxLDL, ng/ml** | 44.7 (6; 854) | 508.9 (27.6; 848.5) | 0.181 |
| **TNF-α, pg/ml** | 37.1 (7.9; 627.7) | 25.8 (10; 80.4) | 0.704 |
| **TGF-β1, pg/ml** | 570.8 (0; 708.7) | 578.2 (0.4; 728.7) | 0.589 |

Data are expressed as median (25th–75th percentile). Pts, patients; RANKL, receptor activator of nuclear factor kappa-B ligand; MGP, matrix Gla protein; hs-CRP, high sensitivity C-reactive protein; oxLDL, oxidized low density lipoprotein; TNF, tumor necrosis factor-α; TGF, tumor growth factor; AU, Agatston units. Significant p values are in boldface.

Table B. Clinical characteristics and metabolic parameters in patients with myocardial infarction in the acute and subacute phases.

| **Pts. with MI (n = 40)** | **Acute Phase MI** | **1-2 months post-MI** | **p** |
| --- | --- | --- | --- |
| **Medications** |  |  |  |
| ACE inhibitor/AT-1 receptor blocker (%) | 27 (67.5) | 24 (60) | 0.508 |
| Aspirin (%) | 40 (100) | 39 (97.5) | # |
| Metformin (%) | 9 (22.5) | 8 (20) | >0.999 |
| Statin (%) | 40 (100) | 39 (97.5) | # |
| Ezetimibe (%) | 3 (7.5) | 4 (10) | >0.999 |
| Fibrate (%) | 1 (2.5) | 1 (2.5) | >0.999 |
| ADP antagonist (%) | 40 (100) | 39 (97.5) | # |
| High-intensity statins | 23 (57.5) | 25 (62.5) | 0.727 |
|  |  |  |  |
| **Laboratory tests** |  |  |  |
| Creatinine, mg/dL | 0.9 (0.2) | 0.9 (0.2) | 0.46 |
| Total cholesterol, mg/dL | 159.9 (38.9) | 133.3 (36.3) | **<0.001** |
| HDL-cholesterol, mg/dL | 39 (12.4) | 43 (12.2) | **0.003** |
| LDL-cholesterol, mg/dL | 92 (31.1) | 62.8 (20.6) | **<0.001** |
| Triglycerides, mg/dL | 150.3 (100.9) | 148.8 (103.4) | 0.615 |
| Glycated hemoglobin, % | 6.1 (1) |  |  |

Data are expressed as mean ± standard deviation (SD), median (25th–75th percentile), or number (%). Pts, patients; MI, myocardial infarction; ACE, angiotensin converting enzyme; ARB, angiotensin II receptor blocker., ADP, adenosine diphosphate receptor; HDL, high density lipoprotein; LDL, low density lipoprotein. Significant p values are in boldface.
